# Development of peptide biosensor for the detection of dengue fever biomarker, nonstructural 1

**DOI:** 10.1371/journal.pone.0222144

**Published:** 2019-09-25

**Authors:** Ji Hong Kim, Chae Hwan Cho, Myung Yi Ryu, Jong-Gil Kim, Sei-Jung Lee, Tae Jung Park, Jong Pil Park

**Affiliations:** 1 Department of Pharmaceutical Engineering, Daegu Haany University, Gyeongsan, Republic of Korea; 2 Department of Chemistry, Institute of Interdisciplinary Convergence Research, Research Institute of Halal Industrialization Technology, Chung-Ang University, Heukseok-ro, Dongjak-gu, Seoul, Republic of Korea; Consiglio Nazionale delle Ricerche, ITALY

## Abstract

Dengue virus (DENV) nonstructural 1 (NS1) protein is a specific and sensitive biomarker for the diagnosis of dengue. In this study, an efficient electrochemical biosensor that uses chemically modified affinity peptides was developed for the detection of dengue virus NS1. A series of amino acid-substituted synthetic peptides was rationally designed, chemically synthesized and covalently immobilized to a gold sensor surface. The sensor performance was monitored via square wave voltammetry (SWV) and electrochemical impedance spectroscopy (EIS). Potential affinity peptides specific for NS1 were chosen according to the dynamic current decrease in SWV experiments. Using circular dichroism, the molar ellipticity of peptides (DGV BP1–BP5) was determined, indicating that they had a mostly similar in random coil structure, not totally identical. Using SWV, DGV BP1 was selected as a promising recognition peptide and limit of detection for NS1 was found to be 1.49 μg/mL by the 3-sigma rule. DGV BP1 showed good specificity and stability for NS1, with low signal interference. The validation of the sensor to detect NS1 proteins was confirmed with four dengue virus culture broth (from serotype 1 to 4) as proof-of-concept. The detection performance of our sensor incorporating DGV BP1 peptides showed a statistically significant difference. These results indicate that this strategy can potentially be used to detect the dengue virus antigen, NS1, and to diagnosis dengue fever within a miniaturized portable device in point-of-care testing.

## Introduction

Dengue fever is a one of the major disease that inflicts a heavy global public health burden [1–3]. Over the past years, the appearance of dengue fever derives from mosquito-borne virus, and it can spread globally into many countries with a tremendous increase in epidemic and endemic proportions [4]. Clinical symptoms and illness showing dengue-induced shock syndrome appear rapidly after viral infection [2, 3, 5]. There are four serotypes (DENV type 1–4), and these are genetically related to other flaviviruses, such as hepatitis C virus, Zika virus and West Nile virus[]. DENV is relatively small in size (40–60 nm), with spherical particles and an isometric nucleocapsid [5]. It contains a single RNA consisting of structural and non-structural proteins [1, 5, 6]. Some reports suggest that the glycoprotein, nonstructural 1 (NS1), is a useful biomarker of infection because of its release and high level accumulation at concentrations up to 50 μg/mL in dengue patients [6, 7]. In the clinic, serum NS1 levels range from 0.04 to 2 μg/mL in patients with primary DENV infection and from 0.01 to 2 μg/mL in those with secondary infection [8, 9]. Therefore, a number of commercial rapid kits for DENV NS1 detection have been developed and characterized [3, 8, 10–13]. Combining NS1 with IgM and IgG can improve the sensitivity and specificity of DENV detection, compared to NS1 alone [8, 14, 15]. However, these methods rely on polyclonal or monoclonal antibodies and involve multistep sample preparation [14]. Direct viral culture or gene-based detection methods used widely, but they are relatively long process for whole procedure and require expensive analytical instruments [5, 16]. Serological assays are also routinely used to confirm definite evidence of infections but these assays are less specific [17]. Thus, there is a need for highly sensitive and specific detection of DENV.

Considering these circumstances, several groups have developed new biosensor system for dynamic NS1 quantification [1, 11, 18, 19]. Immunosensors using fluorescent nanoparticles [19–23], surface plasmon resonance (SPR) methods [24], DNA-based bioassays [3, 18, 25–27], and electrochemical detection methods [5, 12, 28–30] have been reported recently. For example, electrochemical detection is widely used in the developing of label-free and portable point-of-care biosensors [5, 28, 29]. It is conceivable that this is a promising and cost-effective method because of quick response, reliability and low maintenance cost [5, 26, 27, 31, 32, 33]. As described in the literatures, the antibodies required for current immunoassays relatively expensive, especially for multiplexed assays or the testing of multiple samples [1, 5, 16, 27, 34, 35]. Therefore, unique peptides are becoming increasingly popular as effective affinity reagents, in place of antibodies, in the development of new biosensors. Due to their small size and cost effectiveness, they have been used in the creation of new biosensing platforms [5]. One of the most interesting benefits is that they can easily synthesize via customized chemical procedure to make linear or cyclic forms in the small molecular level [5, 36]. In addition, they can easily incorporate to various materials, such as gold, other nanoparticles, and liposomes, for the functionalization and creation of biosensors [11, 16, 18].

Evolutionary phage display is a well-characterized method for screening unique affinity peptides that specifically bind targets of interest [32, 35]. Using this approach, our group recently reported a new electrochemical phage-based sensor for the detection of various targets [37–40]. Therefore, free peptides, not associated with whole M13 phages, may utilize as alternative recognition reagent for the creation of a peptide-decorated electrochemical sensor for the early diagnosis of virus infection or target of interest [38, 39]. Therefore, affinity peptides can act as specific recognition element over antibodies in point-of-care testing because of their structural versatility, specificity and high binding affinity for targets [40]. In this study, five derivatives of synthetic peptides were rationally designed, chemically synthesized and observed their structural insights by circular dichroism. And then, the performance of affinity peptide-decorated electrochemical sensor for the detection of DENV NS1 was evaluated.

## Materials and methods

### Chemical reagents

Recombinant DENV NS1 proteins (DENV-1, DENV-2, DENV-3 and DENV-4, >95% purity) was purchased from Abcam (Cambridge, UK). 1-ethyl-3-(3-dimethylaminopropyl) carbodiimide hydrochloride (EDC, 98%), N-hydroxysuccinimide (NHS, 98%), and 11-mercaptoundecanoic acid (MUA) were purchased from Sigma-Aldrich (St. Louis, MO, USA). Bovine serum albumin (BSA) was also purchased from Sigma-Aldrich. As shown in S1 Table, synthetic peptides modified with a cysteine and a flexible linker were chemically synthesized by Peptron (>95% purity, Daejeon, Korea). PBS buffer (pH 7.4) was used to make NS1 proteins and synthetic peptide stock solutions for square wave voltammetry (SWV) measurements. Unless otherwise noted, all reagents were analytical grade.

### Preparation of dengue virus culture broth

To separate the culture supernatant from the lower layer of DENV particles, host cell precipitation was performed by centrifugation at 3,500 × g for 15 min at 4°C. Then, 50 μL of 40% (w/v) polyethylene glycol (M.W: 8000) in 2.5 M sodium chloride was added to the lower layer of DENV particles and the sample was incubated at 4°C for 8 h. Viral precipitation was performed by centrifugation at 8,000 × g for 15 min at 4°C and the supernatant, without DENV, was obtained for subsequent experiments. Further details of the virus propagation, titration and other preparation method, including quality testing by reverse transcription loop-mediated isothermal amplification method, are described in a previous study [16, 25].

### Circular dichroism (CD) spectroscopy

CD analysis of all synthetic peptides (50 μM) were performed on a CD spectrometer (JASCO, Tokyo, Japan) connected with Spectra Manager version 2 CD Multivariate SSE program at 25°C, using 0.1-cm path length of UV cell in a 1X phosphate-buffered saline (PBS) solution. CD measurements were running 4 times for every sample and the spectrum was monitored, as previously reported [5].

### Functionalization of gold electrode

The Au substrate was prepared by evaporation 200 nm gold on clean silicon wafer (two-inch size), pre-coated with a 20 nm titanium (National NanoFab Center, Daejeon, Korea). The Au-coated substrate was then cut into 1 cm × 2 cm surfaces for further functionalization. All reagents and surfaces were freshly prepared immediately before use. The gold substrates were placed in piranha solution (H_2_O_2_:H_2_SO_4_, 7:3 v/v) to remove residual substances for 5 min followed by distilled water and dried under nitrogen flow, and then immersed in ethanol solution of 1-mercaptoundecanoic acid (MUA) (5 mM) for overnight. Excessive MUA was removed by washing with ethanol. Finally, activated gold substrates were dried under nitrogen flow for further activation. MUA-activated gold substrates were immersed in a 2 mL solution of EDC (400 mM) and NHS (100 mM, 1:1, v/v) in methanol for 2–3 h, according to a previously reported protocol [41]. Activated gold substrates were washed in methanol and then immersed in PBS.

### Binding affinity test on a functionalized gold electrode

**Fig 1:**
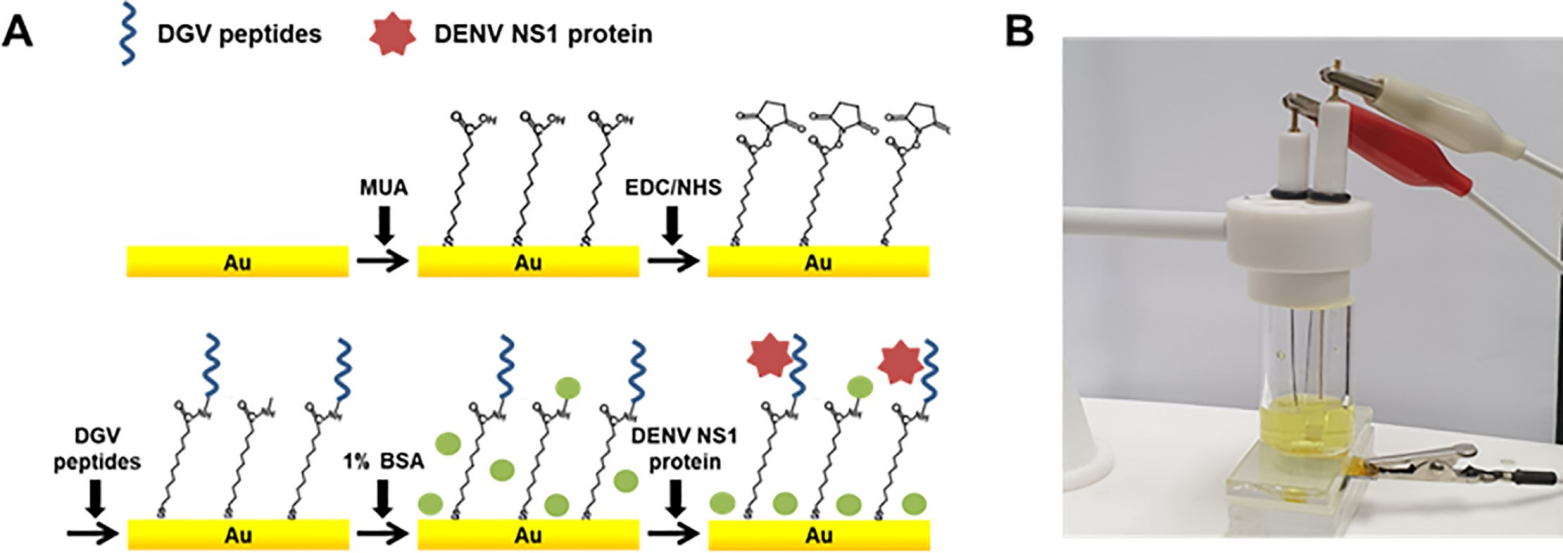
Schematic illustration of the peptide-decorated electrochemical sensor for the detection of dengue fever biomarker, NS1. The functionalized gold surface was exposed to a solution of synthetic peptides to create the affinity peptide-decorated electrochemical sensor for the detection of the dengue virus antigen, nonstructural 1 (NS1).

After preparation and functionalization, the electrodes were assembled and 100 μL of synthetic peptides (10 μg/mL) were dropped onto the gold surface, and thereafter it was incubated at 25°C for 1 h. To remove unbound synthetic peptides, the electrode was washed with PBS buffer and subsequently with distilled water. Then, 1% BSA as blocking agent was placed on the electrode to minimize non-specific interaction. Next, pure DENV-2 NS1 protein (12.5 μg/mL) or dengue virus culture broth derived from four DENV serotypes was placed onto each assembled electrode and then reacted for 1 h at 25°C. Finally, the activated gold electrodes were again sequentially washed with PBS and distilled water to remove unbound protein. The procedure used for the covalent immobilization of synthetic affinity peptides onto a gold surface is described in Fig 1.

### SWV and EIS measurements

The three-electrodes, including a working electrode, platinum counter electrode, and reference electrode, were used for electrochemical measurements. SWV measurements were performed using an electrochemical analyzer (CH Instruments, Austin, TX, USA) connected with data analysis software. These analyses were conducted in a PBS solution containing ferro/ferricyanide (4 mM) as electron mediator according to the previous study [5, 39] using the following conditions: amplitude 5 mV; potential range –0.8 to +0.8 V; scan increment 4 mV and frequency 10 Hz. EIS was carried out at 0.2 V DC potential using 10 mV alternating voltage in the frequency ranging from 10 kHz to 10 Hz.

### Statistical analysis

Two-way ANOVA was used to analyze the cross-reactivity of the binding peptides and the effects of the crude culture broth (GraphPad Software Inc, La Jolla, CA, USA). A Student’s t-test was used to compare relative current changes (Δ*I*%) at the electrode surface. Statistical significance between all cases was determined by ANOVA, where *p* < 0.001.

## Results and discussion

### Rational design and synthesis of affinity peptides

Our preliminary results indicated that phage-displayed peptides were specific for NS1 [5], we assumed that free peptides, separated from the M13 phages, may be suitable to use for the creation of an electrochemical sensor for the efficient detection of DENV antigens, NS1. One limitation of the previous study is that the interaction of the peptides-displayed on M13 phage particles with NS1 targets may be different when affinity peptides are free in solution or when affinity peptides are immobilized on a surface with varying densities. Thus, it needs to be defined and controlled their binding affinity and performance in order to use in practical application. In addition, our results also supported that electrochemical approach should be possible to provide label-free and cost-effective platform in point-of-care testing. To evaluate our hypothesis, five synthetic peptides were rationally designed and chemically synthesized to make covalent immobilization on a gold chip surface. The amino acid sequences and the characteristics of the peptides are shown in S1 Table.

First, the DGV BP1 peptide (EHDRMHAYYLTRGGGGSC), which was identified from phage display, was used as a molecular unit to progressively design other peptide analogs. Second, the DGV BP2 peptide (RTLYYAHMRDHEGGGGSC) was synthesized by reversing the sequence of DGV BP1 and was used to evaluate the roles of the random coil and the extended helical region on binding interactions. Third, the DGV BP3 peptide (EHDRMHAYYLTRGGGGSGGGGSC) was designed with two repeats of a flexible linker (-GGGGS-), to investigate the effects of molecular flexibility on binding interactions. To examine the binding affinity of a peptide with flexibility and a non-fouling nature, the DGV BP4 peptide (EHDRMHAYYLTREKEKEKEGGGGSGGGGSC) was synthesized by incorporating the non-fouling peptide [42], EKEKEKE and two repeats of the flexible linker, GGGGS. Finally, the importance of the molecular unit and length was also investigated using the DGV BP5 peptide (EHDRMHAYYLTREHDRMHAYYLTRGGGGSC), which was made with two repeats of the DGV BP1 sequence.

### Analysis of the secondary structure of synthetic peptides

To analyze the secondary structures of the synthetic peptides and predict their effects on binding interactions, the five analogs of DGV peptides were analyzed using Peptide 2.0 (https://www.peptide2.com) and the Bachem peptide calculator (http://www.bachem.com). DGV BP1 was shown to be rich in basic and hydrophobic uncharged residues (two basic His residues and Arg residues and uncharged hydrophobic residues, including Met, Ala, and Leu). The hydrophilic residue content was 28% and the pI value of the peptide was predicted to be approximately 7.3. DGV BP2 showed an even distribution of acidic, basic, and hydrophobic residues. The pI value of DGV BP2 was predicted to be approximately 7.3. For DGV BP3, the hydrophilic residue content was approximately 26% and its pI value was approximately 7.3. DGV BP4 was rich in acidic and basic residues, with a hydrophilic residue content of approximately 43%. The pI value of DGV BP4 was 6.1. Finally, DGV BP5 was found to be rich in basic and hydrophobic residues, with a predicted pI value of approximately 7.5. The hydrophilic residue content of DGV BP4 was 30%.

**Fig 2:**
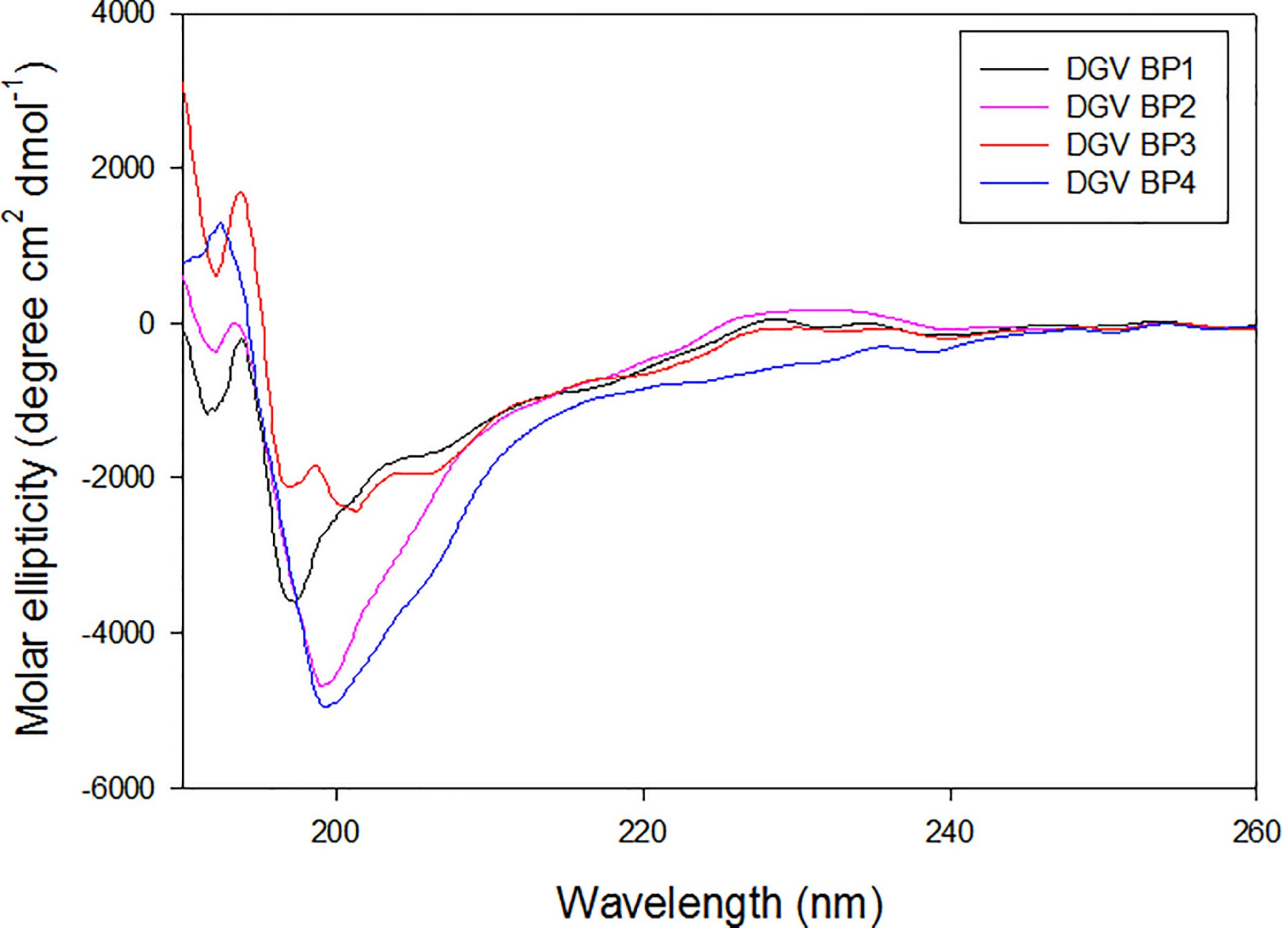
Secondary structure analysis of synthetic affinity peptides used in this study. Circular dichroism (CD) spectra of synthetic peptides (DGV BP1–5), at a concentration of 50 μM, were recorded on a JASCO J-1500 CD spectrometer.

The molar ellipticity of DGV BP1–BP4 peptides were observed similar, but not identical, random coil structures in all five peptides tested (Fig 2).

The CD spectrum of DGV BP1 is indicative of double strong negative band near 190 nm and 197 nm, respectively. In contrast, the CD spectrum for DGV BP2 showed strong negative band near 200 nm. In addition, it was observed weak positive band between 225 nm and 235 nm. In case of DGV BP3, a strong positive band near 195 nm was observed. The CD spectrum of DGV BP4 showed the strongest band near 200 nm and maintained stronger negative band above 200 nm. Secondary structural analysis showed that DGV BP1 was 3.6% α-helices, 40.7% β-sheets, and 13.7% β-turns, while DGV BP2 was 3.8% α-helices, 40.2% β-sheets, and 13.9% β-turns. DGV BP3 consisted of 4.4% α-helices, 40.6% β-sheets, and 13.7% β-turns and DGV BP4 was 4.8% α-helices, 39% β-sheets, and 13.8% β-turns. Unfortunately, no CD spectra of DGV BP5 were obtained due to the residual salts in the sample.

### Selection of potential synthetic peptides for dengue virus NS1 detection

**Fig 3:**
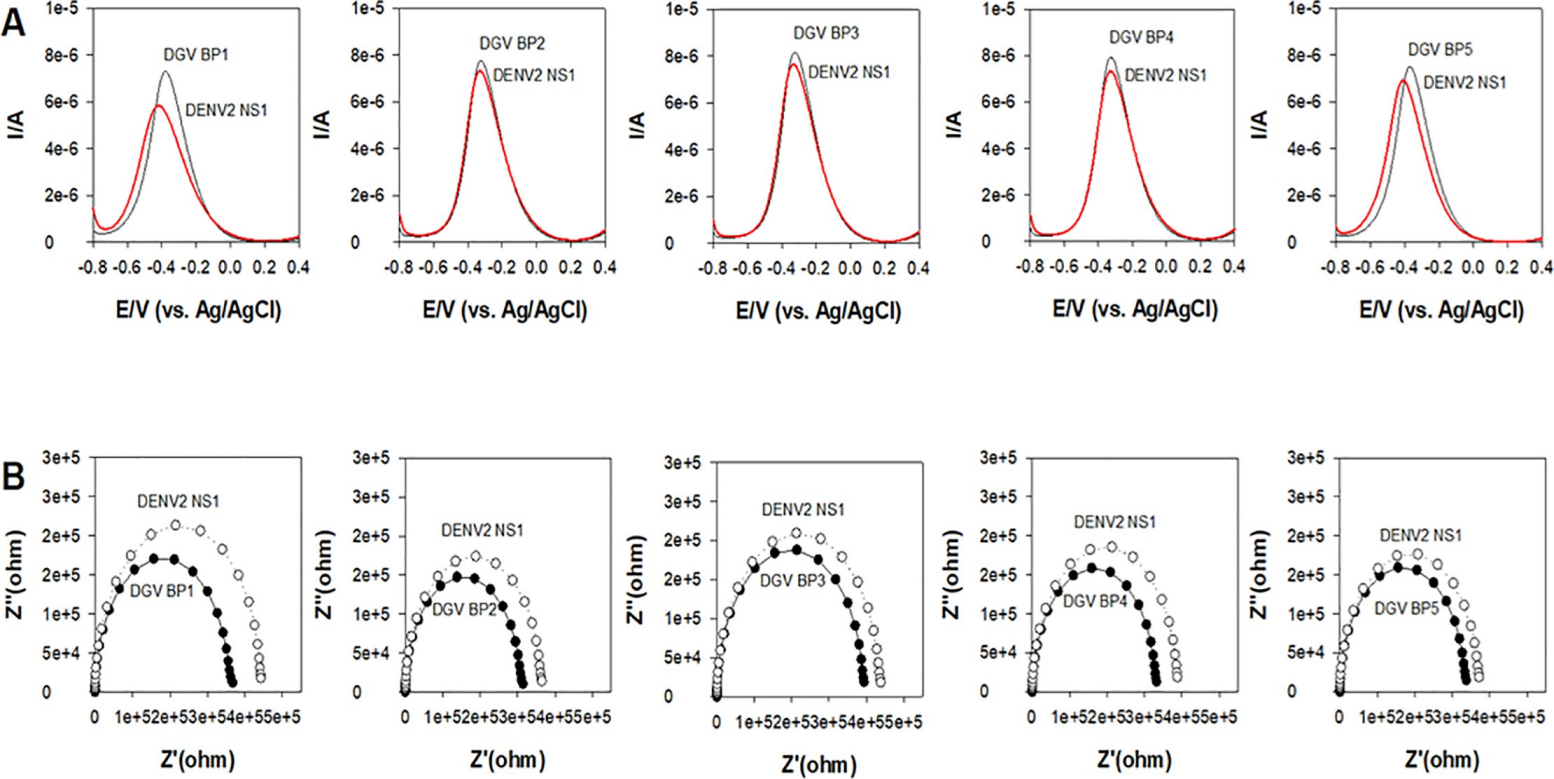
Relative binding affinities of five synthetic peptides (DGV BP1–5), determined by square wave voltammetry (SWV) measurements of dengue virus (DGV) type 2 nonstructural 1 (NS1) proteins (denoted DENV2 NS1, 12.5 μg/mL). A series of synthetic peptides were immobilized onto a gold surface and the current change was measured by SWV.

To determine the best synthetic peptides for DENV NS1 protein detection, the relative binding affinities of the five peptide analogs (DGV BP1–BP5) were compared using SWV measurements (Fig 3).

The relative binding affinities of the peptides were measured by comparing the interactions of NS1 on a pre-immobilized gold surface. Binding was measured as a dynamic change in current using SWV measurements. Among the peptides tested, DGV BP1 resulted in the greatest decrease in current change. When current change was monitored before and after NS1 protein binding, a linear correlation was observed between the current and the presence of protein, due to mass loading and specific recognition of NS1. The binding affinities of the five peptides followed the order, DGV BP1 > DGV BP5 > DGV BP4 > DGV BP3 > DGV BP2. We confirmed that this is consistent to EIS measurements. These results indicated successful covalent immobilization of the peptides and specific binding of NS1 proteins on the gold surface, providing high affinity for NS1. We also checked binding interaction on a non-functionalized gold sensor layer. It was observed that change in impedance was not correlated with NS1 binding, indicating that no direct covalently immobilization of synthetic peptides on a gold surface (Fig A in S1 File). Based on these observations, we chose DGV BP1 as a promising peptide for detection of DENV-2 NS1. It is noteworthy that DGV BP1 peptide enables to form random coil structure and was rich in basic and hydrophobic uncharged residues. It was made up of 28% hydrophilic residues and had a predicted pI value of approximately 7.3. These structural features suggested that number of basic and hydrophobic residues, along with the pI value, may be the dominant factors affecting the binding of NS1.

### Effect of DGV BP1 peptide concentrations on binding interactions

After selecting the potential peptide, we next used SWV measurements to investigate the effects of DGV BP1 peptide concentration on binding to NS1. The dynamic peak response at varying DGV BP1 concentrations (from 1 to 100 μg/mL) was measured before and after reaction with NS1 (12.5 μg/mL). The decrease in current was calculated based on the relative current change (Δ*I*%), considering the peak current values of the SWV voltammograms obtained after covalent peptide immobilization and protein incubation. Calculations were made using the following equation [41]:

ΔI%=(Ib−Ia)/Ib×100, (Eq 1)

where Δ*I* is relative current change (%) and *I*_*b*_ and *I*_*a*_ represent the current change before and after protein incubation, respectively.

**Fig 4:**
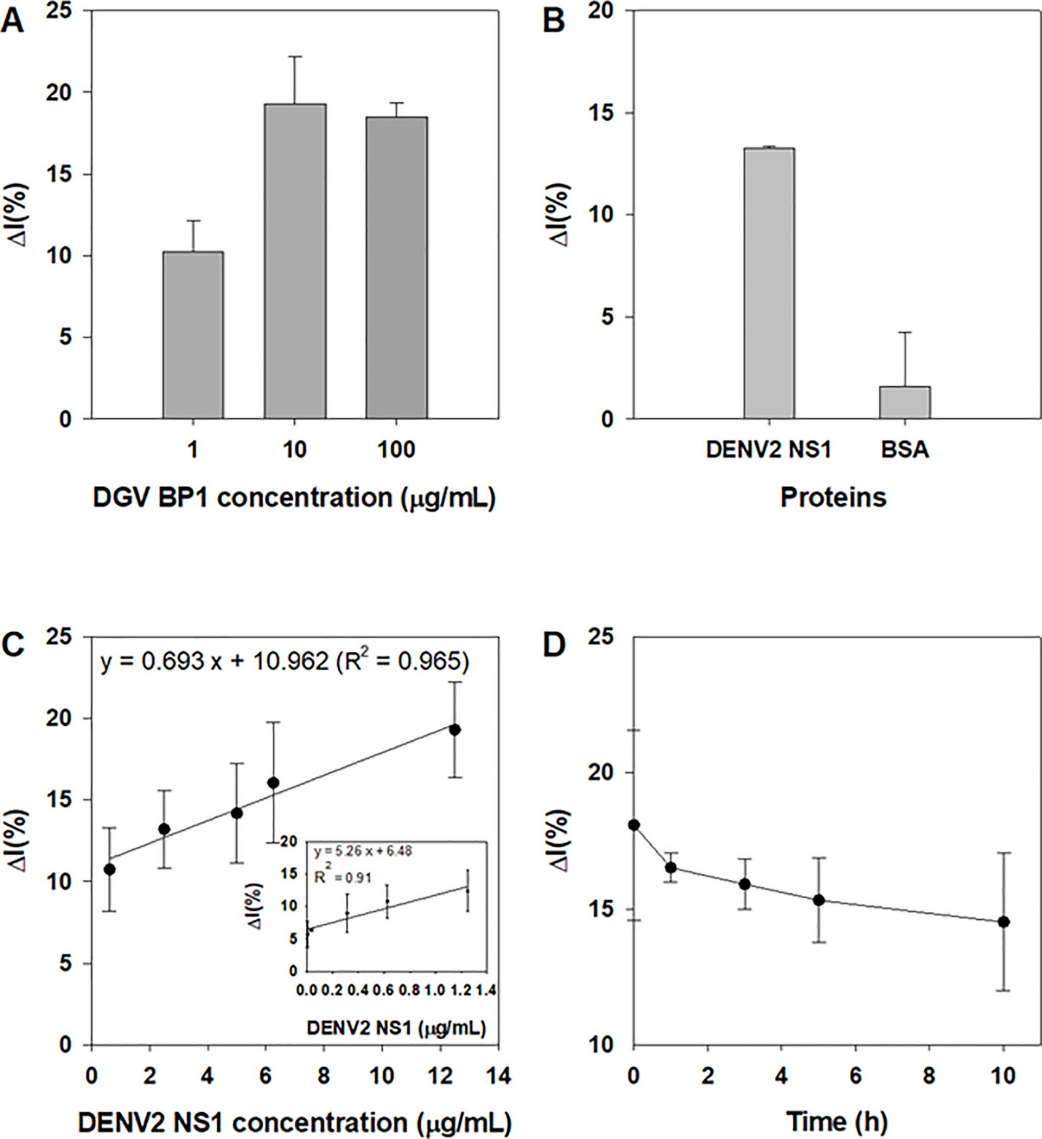
Relative binding affinity. (A) Effect of peptide concentrations. The current change at varying concentrations of DGV BP peptides (from 1 to 100 μg/mL) on a gold electrode layer was measured before and after the addition of pure dengue virus (DENV) type 2 nonstructural 1 (NS1) proteins (denoted DENV2 NS1, 12.5 μg/mL). (B) Relative current change on the binding interaction. (C) Linear correlation of the sensor with different NS1 protein concentrations, measured by square wave voltammetry. Inset shows relationship between relative *I*(%) with lower concentration of NS1 ranging from 0 to 1.4 μg/mL. (D) Stability test of affinity peptide-decorated sensor.

The Δ*I*% value increased with increasing concentrations of DGV BP1 peptides up to 10 μg/mL and reached saturation at 100 μg/mL. Therefore, a DGV BP1 peptide concentration of 10 μg/mL was selected as optimum concentration for subsequent experiments (Fig 4A).

### Specificity, sensitivity and stability testing of the sensor system

The specificity of the sensor was tested after immobilization of DGV BP1 peptides on a gold electrode and subsequent treatment with NS1 or BSA. DGV BP1 peptides were immobilized on a gold surface at 25°C and washed with PBS buffer. Peptide-immobilized electrodes were then incubated for 3 h at room temperature. After incubation, pure DENV-2 NS1 and BSA, which was used as a control at the same concentration (12.5 μg/mL), were separately added onto the peptide-immobilized electrode and Δ*I*% value was measured. The Δ*I*% value was much higher for NS1 than for BSA (Fig 4B and Fig B in S1 File). The sensitivity of the sensor system was evaluated by taking into account the Δ*I*% values for different NS1 concentrations. As seen in Fig 4C, Δ*I*% values were highly correlated with NS1 concentration (regression coefficient, R^2^ = 0.965), with Δ*I*% values increasing with increasing NS1 concentrations from 0.003 to 12.5 μg/mL. As shown in Fig C in S1 File, the limit of detection (LOD) was calculated by the following equation LOD = 3 (σ/*S*) [43], where, σ is the standard deviation and *S* is the sensitivity and LOD of our sensor system was found to be 1.49 μg/mL which is slightly lower or comparable to that of the previously reported biosensor [1, 44]. However, it is noteworthy that LOD value in our sensor is close to the level of NS1 in primary (0.04 to 2 μg/mL) and secondary DENV infection (0.01 to 2 μg/mL) in patients [8, 9] suggesting quite acceptable for clinical testing. Importantly, a strong linearity with good correlation (regression coefficient R^2^ = 0.91) between Δ*I*% and NS1 concentration was seen in ranging from 0.003 to 1.25 μg/mL (inset in Fig 4C). Even though LOD of this sensor is slightly higher compared with immunoassay, the use of chemically modified or structurally rational designed affinity peptide can be improved binding affinity and sensitivity. Because one of the most exciting advantages of the affinity peptides is relatively small in size and their cost-effectiveness in mass production compared to those of antibody production [37, 39]. Moreover, they are relatively more stable than antibodies and can easily be applied into point-of-care sensor system for clinical applications. We confirmed that DGV BP1 peptides were comparable to NS1 the previously reported LOD values, for example, 0.03 μg/mL with electrochemical immunosensor [44] and 20 ng/mL-20 μg/mL with optomangnetic method [1)). We found that this system was sensitive for NS1 in the development of dengue biosensor. In order to assess the stability of the sensor, DGV BP1-tethered electrodes (10 μg/mL of DGV BP1) were incubated with NS1 protein (12.5 μg/mL) at room temperature for up to 10 h. The change in current was then measured using SWV from 1 h to 10 h. Our sensor was found to be stable for 10 h, with only a 3.56% loss in the initial Δ*I*% (18.07) over that time period (Fig 4D).

### Validation of sensor performance with dengue virus culture broth

**Fig 5:**
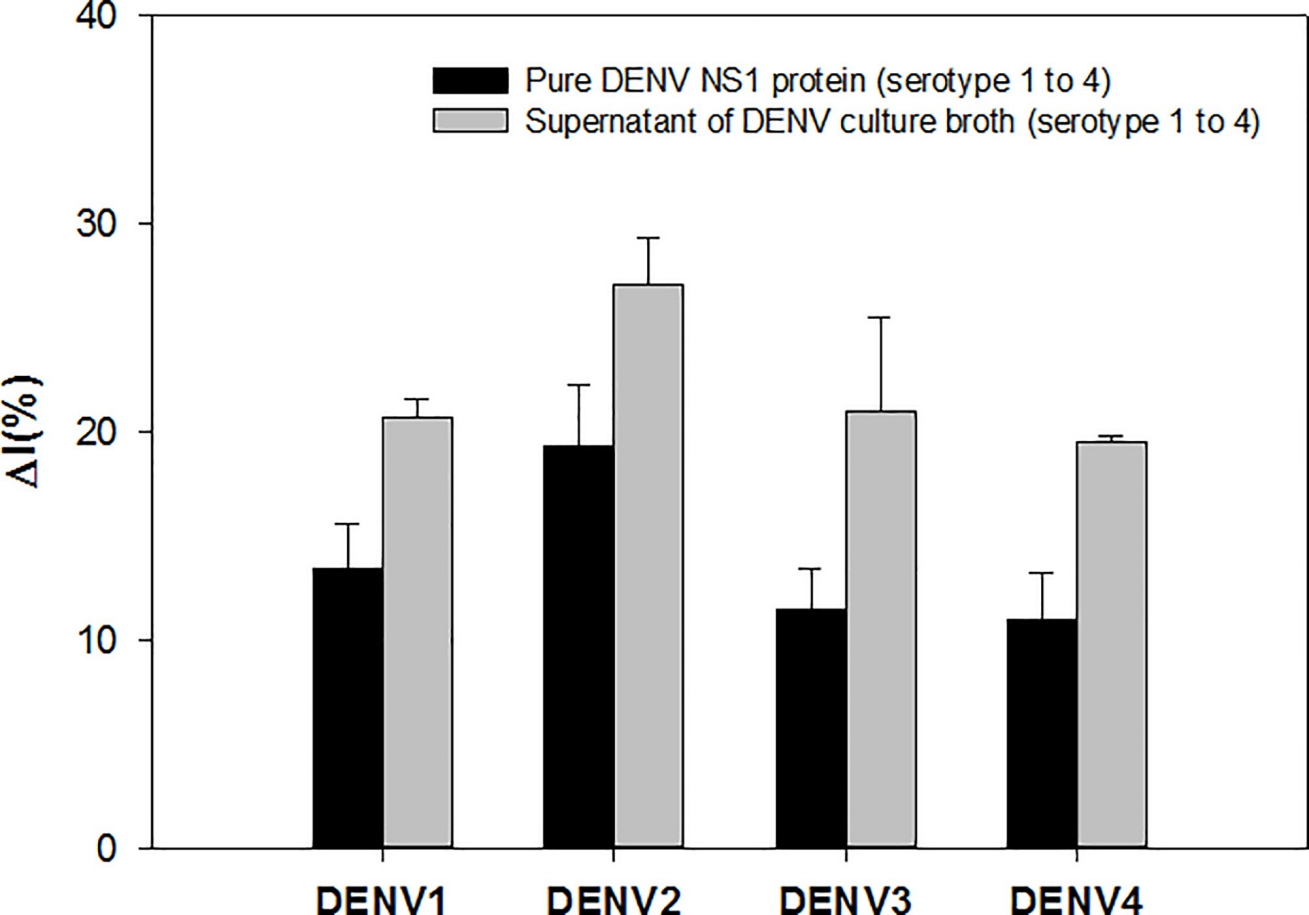
Relative binding affinity of DGV BP1 peptides with dengue virus (DENV) culture broth (serotype 1; DENV1, type 2; DENV2, type 3; DENV3, type 4; DENV4). Current change was measured by square wave voltammetry before and after the addition of four pure DENV nonstructural 1 (NS1) proteins (12.5 μg/mL) and culture broth from DENV serotypes 1–4 to a DGV BP1 peptide-decorated gold electrode layer.

To study the feasibility of the sensor, SWV was used to measure relative current change after the addition of pure NS1 proteins and culture broth derived from DENV serotype 1–4, respectively. As shown in Fig 5, the current change in the presence of pure NS1 (type 2) protein was much higher than the current change in the presence of other pure NS1 proteins (type 1, type 3–4). This result suggested that free DGV BP1 was highly specific and selective for binding to DENV-2 NS1.

To further test the selectivity of the sensor in a biological matrix, SWV signals were recorded in culture broth samples from varying DENV serotypes (type 1–4). Compared to pure NS1 proteins, Δ*I*% values were much higher for culture broth samples. This may be due to the complicated biological matrix of culture broth, which included various other components that may influence the detection results. However, a statistically significant difference was seen among culture broth samples from the four different DENV serotypes, indicating that the DGV BP1-decorated sensor system was still specific for the detection of NS1 proteins. The repeatability of the sensor was also investigated at the NS1 proteins concentration (12.5 μg/mL). Freshly prepared gold electrode with DGV BP1 was used for the detection of NS1 and changes in current for 4 cycles were monitored in SWV measurements (Fig D in S1 File). It was observed that our sensor with DGV BP 1 peptide was stable up to third regeneration with good reproducibility. Despite this observation, our peptide-assisted electrochemical sensor needs to be elucidating broad selectivity with NS1 derived from other flaviviruses, such as Spondweni, Yellow fever and Zika virus. Understanding these observations, we conclude that affinity peptide-decorated electrochemical sensor may be adaptable to a simple and cost-effective miniaturized microdevice detection kit to monitor DENV infections.

## Conclusions

In this study, we demonstrated a novel method for the creation of an affinity peptide-decorated electrochemical biosensor, in which synthetic affinity peptides were tethered to an gold electrode surface for detection of the DENV antigen, NS1. This process included the selection of a peptide for target, rationally design and synthesis of the affinity peptide (DGV BP1–BP5), structural study, creation of the sensor, and detection of NS1 by electrochemical techniques (SWV and EIS). Among all the peptides tested, DGV BP1 was selected as a potential affinity peptide for our sensor system. It showed the greatest current decrease in SWV measurements and dynamic impedance increase in EIS measurements, indicating specific recognition and binding, with a strong linear correlation between DGV BP1 and NS1 protein concentrations. To the best our knowledge, this is the first example of rational design, synthesis and characterization of DENV NS1-binding affinity peptides for detection of dengue virus antigen using electrochemical methods (SWV and EIS measurements). The developed electrochemical biosensor showed high sensitivity, stability, and specificity, being able to detect and quantify NS1 within the concentration ranges usually reported in patient plasma samples. The ability of the sensor to detect NS1 proteins in DENV culture broth demonstrated significant difference (*p* < 0.001) in different DENV serotypes (type 1–4).

Our sensor system had much higher specificity for NS1 derived from DENV-2 than NS1 from other DENV serotypes. Based on these observations, this strategy is straightforward and cost-effective for the development of a sensor for point-of-care testing, compared with conventional antibody-based immunoassay, and can be applied for the detection of NS1 from various DENV serotypes or other flaviviruses. Further evaluation of this technology using human blood or serum from dengue virus infected patients is under consideration. In addition, the presence of many other proteins or small molecules in blood or serum may interfere with detection and this is also underway.

## Supporting information

S1 Table Characteristics of the synthetic peptides used in this study. (DOCX)

S1 File Comprises: Electrochemical response using non-functionalized gold electrode (Fig A); Selectivity test of DGV BP1 peptide (Fig B); Calculation of limit of detection (Fig C); Repeatability test (Fig D).
(DOCX)
