# Supplementary material for: Development of peptide biosensor for the detection of dengue fever biomarker, nonstructural 1
Source: PLoS One. 2019 Sep 25;14(9):e0222144. doi: 10.1371/journal.pone.0222144 (PMC6760828; doi:10.1371/journal.pone.0222144)
Supplement: S1 Table — (DOCX) [file pone.0222144.s001.docx]

**S1 Table. Characteristics of the synthetic peptides used in this study.**

| Name | Amino acid sequence | Mass analysis  (MS found) | Predicted pI | Notes |
| --- | --- | --- | --- | --- |
| DGV BP1 | EHDRMHAYYLTRGGGGSC | 2010 | 7.3 | -Selected upon biopanning  -Used as peptide scaffold for rational design  - Hydrophobic: 16.67%, acidic: 11.11%  basic: 22.22%, neutral: 50% |
| DGV BP2 | RTLYYAHMRDHEGGGGSC | 2009 | 7.3 | -Reverse sequence of DGV BP1  -Hydrophobic: 16.67%, acidic: 11.11%  basic: 22.22%, neutral: 50% |
| DGV BP3 | EHDRMHAYYLTRGGGGSGGGGSC | 2325 | 7.3 | -Two repeats of a flexible linker (-GGGGS-) to investigate the effects of molecular flexibility on binding interactions  -Hydrophobic: 13.04%, acidic: 8.7%  basic: 17.39%, neutral: 60.87% |
| DGV BP4 | EHDRMHAYYLTREKEKEKEGGGGSGGGGSC | 3226 | 6.1 | -Incorporating the non-fouling peptide, EKEKEKE and two repeats of the flexible linker, GGGGS  -Hydrophobic: 10%, acidic: 20%, basic: 23.33%, neutral: 46.67% |
| DGV BP5 | EHDRMHAYYLTREHDRMHAYYLTRGGGGSC | 3583 | 7.5 | -Two repeat of DGV BP1  -Hydrophobic: 20%, acidic: 13.33%, basic: 26.67%, neutral: 40% |
