# Supplementary material for: Development of peptide biosensor for the detection of dengue fever biomarker, nonstructural 1
Source: PLoS One. 2019 Sep 25;14(9):e0222144. doi: 10.1371/journal.pone.0222144 (PMC6760828; doi:10.1371/journal.pone.0222144)
Supplement: S1 File — Comprises: Electrochemical response using non-functionalized gold electrode (Fig A); Selectivity test of DGV BP1 peptide (Fig B); Calculation of limit of detection (Fig C); Repeatability test (Fig D). (DOCX) [file pone.0222144.s002.docx]

**Supporting Information**

Development of Peptide Biosensor for the Detection of Dengue Fever Biomarker, Nonstructural 1

Ji Hong Kim^1¶^, Chae Hwan Cho^1¶^, Myung Yi Ryu ^1^, Jong-Gil Kim ^2^, Sei-Jung Lee ^1^, Tae Jung Park ^2^* and Jong Pil Park^1^*

^1^ Department of Pharmaceutical Engineering, Daegu Haany University, Gyeongsan 38610, Republic of Korea

^2^ Department of Chemistry, Institute of Interdisciplinary Convergence Research, Research Institute of Halal Industrialization Technology, Chung-Ang University, 84 Heukseok-ro, Dongjak-gu, Seoul 06974, Republic of Korea

* Corresponding author.

E-mail: jppark@dhu.ac.kr (JP Park), tjpark@cau.ac.kr (TJ Park)

^¶^ These authors contributed equally to this work.

**Fig A. Electrochemical response using non-functionalized gold electrode.** DGV BP1 peptide (10 μg/mL) was immobilized on bare gold chip and incubated with dengue virus type 2 NS1 (12.5 μg/mL) at room temperature. Impedance changes were measured by EIS measurements.

**Fig B. Selectivity test of DGV BP1 peptide on different proteins (A) and other synthetic peptide (B).** DGV BP1 (10 μg/mL) were incubated with various proteins (BSA, PCT, SA and NS1, 12.5 μg/mL). In the experiment B, NS1 (12.5 μg/mL) was incubated with Noro-1 and DGV BP1 peptides(10 μg/mL) at room temperature. Changes in current and relative binding affinities were measured by using SWV.

* Noro-1 sequence: QHKMHKPHKNTKGGGGSC (Reference: Biosensors and Bioelectronics 87 (2017) 164–170)

**Fig C. Calculation of limit of detection with sensor system.**


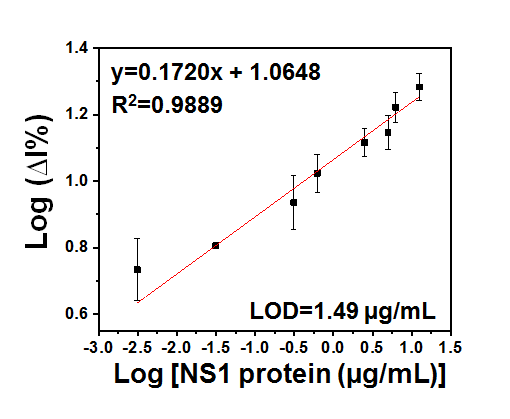


**Fig D. Repeatability and regeneration test of the sensor chip.**
